# Working Memory and Instructional Fit: Reintroducing Aptitude–Treatment Interaction in Education Research

**DOI:** 10.3390/bs15060765

**Published:** 2025-06-02

**Authors:** Faria Sana, Barbara Fenesi

**Affiliations:** 1 Faculty of Humanities and Social Sciences, Athabasca University, Athabasca, AB T9S 3A3, Canada; 2 Faculty of Education, Western University, London, ON N6G 1G7, Canada; bfenesi@uwo.ca

**Keywords:** working memory, academic achievement, aptitude–treatment interaction

## Abstract

Working memory (WM) is a cognitive system with limited capacity that enables individuals to focus on goal-relevant information while filtering distractions and integrating new knowledge. Differences in WM capacity influence how students engage with instructional materials, shaping the effectiveness of educational interventions. This raises the following question: which instructional methods work best, for whom, and under what conditions? The aptitude–treatment interaction (ATI) framework addresses this by examining how learning outcomes are influenced by the alignment between cognitive abilities, such as WM, and instructional strategies. This paper reviews WM’s role in learning and academic achievement, explores ATI’s relevance in contemporary education research, and highlights how this framework can guide the development of adaptive instructional strategies that support diverse learners.

## 1. Introduction

Over the past century, cognitive psychology has significantly advanced our understanding of human learning and memory. Research highlights that learning outcomes are influenced by underlying cognitive abilities, which vary meaningfully among individuals (Rohrer & Pashler, 2010; Dunlosky & Rawson, 2019). Contemporary educational research increasingly acknowledges that the effectiveness of instructional techniques cannot be universally assumed but depends on individual learner characteristics. However, despite broad recognition, cognitive aptitudes such as working memory (WM) remain underutilized in instructional design and practical educational contexts. These abilities are often treated as random variance rather than as key determinants that explain differential instructional effectiveness (Sala & Gobet, 2020). To address this gap, this paper focuses on working memory capacity (WMC) within the aptitude–treatment interaction (ATI) framework, highlighting how aligning instructional strategies with learners’ WMC can significantly enhance learning outcomes.

The ATI framework, originally conceptualized by Cronbach and Snow (1977), seeks to identify instructional treatments whose effectiveness varies systematically based on learner aptitudes—relatively stable cognitive, affective, or motivational characteristics (Preacher & Sterba, 2018; Rohrer & Pashler, 2010). Instructional treatments refer to pedagogical strategies designed to facilitate learning, such as multimedia instruction, retrieval practice, spaced repetition, and feedback provision. An aptitude–treatment interaction occurs when instructional outcomes differ systematically based on learners’ cognitive characteristics (Mayer, 2017). The ATI perspective is grounded in the assumption that learners with different cognitive abilities engage in qualitatively distinct cognitive processes rather than simply learning at different rates (Rohrer & Pashler, 2010). Instructional strategies beneficial for one group, such as individuals with high WMC, may provide limited or even negative outcomes for another group, such as individuals with low WMC. Empirical research supports this assumption, demonstrating that some instructional methods designed to optimize WM resources disproportionately benefit learners with lower WMC without negatively impacting higher-capacity peers (Fyfe & Rittle-Johnson, 2017; Peng & Fuchs, 2016).

Although numerous cognitive traits contribute to learning—such as executive function, attentional control, and fluid intelligence—this paper focuses specifically on WMC for both theoretical and practical reasons. First, WMC is a well-defined, reliably measured construct that plays a central role in managing the encoding, maintenance, and manipulation of information during learning tasks. It is strongly predictive of academic achievement across subject areas, even when controlling for broader intelligence measures (Alloway & Alloway, 2010). Second, WMC supports and overlaps with many other aptitudes, serving as a cognitive bottleneck that affects performance in tasks requiring attention, reasoning, or self-regulation. As such, it offers a conceptually coherent and empirically tractable target for investigating ATI. Finally, unlike broader constructs such as intelligence or executive function, WMC is more easily aligned with specific instructional demands, making it especially well suited for designing personalized, evidence-based educational interventions.

To this end, our paper aims to achieve three primary objectives: (1) to provide a comprehensive theoretical and empirical overview of WM as a cognitive aptitude and its influence on educational performance, (2) to briefly review recent ATI research that examines the interactions between WMC and instructional strategies, and (3) to discuss the key methodological considerations while outlining both the practical and theoretical implications for educators and researchers who seek to apply the ATI framework in educational settings. The structure of the paper follows these objectives.

## 2. WM and Cognitive Performance

The definition, structure, and function of WM have been extensively debated. However, three fundamental aspects are well established in the literature. First, WM is closely linked to attentional control and is not merely a passive storage system. Rather, it plays an active role in keeping task-relevant information accessible while filtering out distractions (Oberauer, 2019; Shipstead et al., 2016). Second, WMC is a relatively stable cognitive trait, meaning that although it can fluctuate depending on factors such as fatigue or motivation, it remains constrained by biological limits and does not change significantly over time (Redick et al., 2016; Xu et al., 2018). Third, deficits in WM significantly impact daily functioning and academic performance, and they are commonly associated with neurodevelopmental disorders such as ADHD and dyslexia (Snyder et al., 2015).

For educational contexts, WM is best understood as comprising two interrelated components: domain-general control processes and domain-specific storage mechanisms. The domain-general aspect of WM includes attentional control and strategic retrieval from long-term memory, which help learners maintain focus on relevant information while suppressing distractions (Shipstead et al., 2016; Unsworth & Robison, 2020). The domain-specific aspect refers to the short-term storage of different types of information, including phonological and auditory input, which are processed in the verbal store, and visual, spatial, and movement-related information, which are handled by the visuospatial store (Logie et al., 2021). These two subsystems work in tandem, linking new information with pre-existing knowledge stored in long-term memory, while domain-general processes ensure that these connections remain active (Cowan, 2017; Logie et al., 2021).

WMC is typically measured using complex span tasks that assess both storage and processing simultaneously. A widely used example is the reading span task, in which participants read sentences while recalling the final word of each. Another common measure is the operation span task, where individuals solve math problems while remembering numerical information. The symmetry span task, which requires recalling visual patterns while making spatial judgments, is also frequently employed (Unsworth et al., 2005; Foster et al., 2015). Despite differences in format, all these tasks assess the same fundamental ability: the capacity to juggle multiple pieces of information while engaging in higher-order cognitive processes. These tasks reliably differentiate between high- and low-WMC individuals, and refinements in measurement techniques have further strengthened their predictive power for academic performance (Draheim et al., 2016; Redick et al., 2016).

Individual differences in WMC are strongly associated with higher-order thinking skills, including reasoning, comprehension, and problem-solving. Reading comprehension, for example, relies on the ability to hold onto words and phrases long enough to integrate meaning across sentences, resolve ambiguities, and draw inferences. Research has consistently shown that WMC is a strong predictor of reading ability, independent of general intelligence and verbal proficiency (Peng et al., 2018). Similarly, in mathematics, WM is essential for multi-step problem-solving, where students must retain intermediate calculations while applying formulas or reasoning strategies (Friso-van den Bos et al., 2015).

Beyond complex academic tasks, WM also plays a fundamental role in basic cognitive functions such as attention control, inhibition, and interference management. Individuals with higher WMC are better at maintaining focus on relevant information while ignoring distractions, which explains why they tend to perform better in cognitively demanding environments (Unsworth & Robison, 2020). In contrast, individuals with lower WMC struggle more with sustained attention and are more susceptible to mind-wandering (Shipstead et al., 2016). WM is also strongly linked to proactive interference, a phenomenon in which previously learned information disrupts the retrieval of new material. Studies indicate that lower-WMC individuals generate retrieval cues that are less distinct, making it more difficult to access relevant information when needed (Unsworth & Engle, 2007; Unsworth et al., 2012b).

One of the most widely studied relationships in WM research is its connection to fluid intelligence (gF)—the capacity to reason abstractly, identify patterns, and solve novel problems (Burgess et al., 2011). Numerous studies have demonstrated a strong correlation between WMC and gF, with neuroimaging evidence indicating that both rely on overlapping brain networks in the frontal and parietal regions, which are crucial for executive function and controlled processing (Burgess et al., 2011; Finn et al., 2015). Individuals with higher WMC tend to exhibit greater neural efficiency, meaning they can perform cognitive tasks with reduced mental effort. This efficiency is particularly evident in tasks that require filtering out irrelevant information or maintaining multiple sources of information simultaneously (Shipstead et al., 2014).

The link between WM, attentional control, and intelligence has significant implications for learning. When instructional materials present high cognitive demands, learners with lower WMC may be more susceptible to cognitive overload, which occurs when task complexity exceeds an individual’s processing capacity. This is particularly problematic in disciplines such as mathematics and science, where students must integrate multiple sources of information simultaneously. If instructional strategies do not account for these limitations, lower-WMC students may struggle to process content effectively, even if they possess sufficient domain knowledge.

In summary, WM serves as a fundamental component of learning, problem-solving, and daily cognitive functioning. Its role in managing attention, suppressing distractions, and integrating new information makes it a critical predictor of academic success (Alloway & Alloway, 2010). Given the broad range of cognitive processes affected by WMC, understanding how instructional strategies interact with WM differences is essential for designing effective educational interventions.

## 3. WM and Academic Achievement

The previous section established the essential role of WM in cognition and learning. This section examines how individual differences in WMC predict academic success across core educational domains, including reading comprehension, writing, mathematics, second language acquisition, and science learning. In addition to subject-specific outcomes, WMC also influences fundamental classroom behaviors such as note-taking, following instructions, and managing distractions. Synthesizing recent research on these relationships highlights the broad impact of WM on both academic achievement and classroom engagement.

### 3.1. Role of WM in Academic Domains

#### 3.1.1. Reading Comprehension

WMC is widely recognized as a key determinant of reading comprehension. Successful reading requires the continuous processing, integration, and updating of textual information. Individuals with higher WMC manage complex and lengthy passages more effectively, particularly when dealing with embedded clauses, ambiguous pronouns, or abstract concepts (Peng et al., 2018; Swanson et al., 2016). Even when controlling for factors such as verbal ability, decoding skills, and attention, WMC remains a strong predictor of reading comprehension outcomes (Kim, 2016). This relationship holds across diverse languages and writing systems, suggesting that WM plays a universal role in reading development. In contrast, lower-WMC readers are more likely to experience comprehension breakdowns, particularly when required to draw inferences or integrate information across multiple sentences. These findings highlight the importance of instructional strategies that support lower-WMC learners by reducing cognitive load in reading tasks.

#### 3.1.2. Written Expression

Writing places substantial demands on WM, as it requires the simultaneous execution of multiple cognitive processes. Writers must generate ideas, structure sentences, maintain coherence, and monitor for grammatical accuracy while holding key elements in WM. Research has shown that WMC is a significant predictor of writing proficiency, particularly in areas such as syntactic complexity, coherence, and mechanical accuracy (Olive, 2014; Vanderberg & Swanson, 2007). Lower-WMC learners often struggle to maintain cohesion in extended writing tasks and are more prone to errors, particularly under time constraints or increased cognitive load. Studies on writing development suggest that transcription fluency and self-regulation are key mediators in how cognitive resources impact writing quality (Limpo & Alves, 2013). These findings reinforce the role of WMC in written expression and highlight the importance of instructional strategies that alleviate cognitive strain during writing tasks.

#### 3.1.3. Second Language Acquisition

Second language acquisition also relies heavily on WMC, particularly in vocabulary learning, syntactic processing, and phonological representation. Research has demonstrated that phonological short-term memory is crucial for acquiring new vocabulary, as higher-WMC learners retain and integrate novel linguistic patterns more effectively. For instance, Baddeley (2003) discusses the role of WM in language learning, highlighting its importance in vocabulary acquisition. Additionally, studies indicate that high-WMC individuals manage syntactic complexity more efficiently and adapt to new language structures with greater ease across different language pairs. Juffs and Harrington (2011) review the role of WM in second language learning and processing, emphasizing its impact on syntactic processing. Similarly, Linck et al. (2014) found that WMC is a significant predictor of language learning success, particularly in managing complex syntactic structures. In contrast, lower-WMC learners require more repetition and structured exposure to achieve comparable proficiency levels. These findings underscore the importance of instructional strategies that reduce cognitive load during second language learning to support learners with lower WMC.

#### 3.1.4. Mathematics and Problem-Solving

Mathematics is another domain where WM plays a central role, particularly in multi-step calculations, algebraic reasoning, and problem-solving. Meta-analyses confirm that WMC is one of the strongest cognitive predictors of mathematical proficiency, particularly in tasks requiring sustained attention, information manipulation, and mental arithmetic (Peng et al., 2016; Zhang et al., 2023). Students with higher WMC are more adept at retaining numerical values, tracking intermediate steps, and applying problem-solving strategies efficiently. Conversely, those with mathematical learning difficulties frequently exhibit lower WMC, which affects their ability to retrieve math facts, maintain WM representations, and process multi-step problems (Geary et al., 2017; Fung & Swanson, 2017). These effects are especially pronounced in abstract problem-solving tasks that require learners to juggle multiple pieces of information without external aids.

#### 3.1.5. Science Learning and Reasoning

Science learning often involves complex cognitive tasks such as reasoning through causal relationships, analyzing experimental data, and interpreting scientific texts. These tasks require the integration of multiple information sources simultaneously, including textual explanations, diagrams, and data representations. Research indicates that students with higher WMC may perform better in problem-solving environments, as they can manage and coordinate multiple information streams more effectively. For instance, Wiley and Jarosz (2012) discuss the relationship between WMC and problem-solving abilities, highlighting that higher WMC can be beneficial in analytical problem-solving contexts. Similarly, Tolmie et al. (2016) explore the role of executive functions, including WM, in children’s science learning, suggesting that these cognitive skills are foundational for developing scientific reasoning abilities. Conversely, students with lower WMC may struggle to synthesize information from various sources, making it more challenging to grasp complex scientific concepts. As science education increasingly incorporates multimodal instruction, understanding how WMC interacts with different instructional formats remains an important area for future research.

While WMC influences a wide range of learning outcomes, this review focuses specifically on academic achievement due to its relevance for instructional design and evidence-based intervention. Importantly, WMC does not operate uniformly across subjects; each domain imposes distinct cognitive demands, which in turn affect how ATI-informed strategies should be applied. For example, lower-WMC learners may benefit more from scaffolding and worked examples in mathematics, while segmentation and dual-channel multimedia may be better suited to support science learning. In contrast, reading comprehension and second language acquisition often place greater strain on phonological memory, requiring repetition and structured exposure. These differences underscore the importance of tailoring instructional supports not only to cognitive aptitude but also to the demands of the academic subject.

### 3.2. Role of WM in Classroom Skills

Beyond subject-specific outcomes, WMC also affects essential classroom behaviors, including reasoning, participation, note-taking, and behavior regulation. While these skills are often overlooked in traditional assessments of academic achievement, they significantly influence learning outcomes and engagement.

#### 3.2.1. Classroom Reasoning and Participation

Inferential reasoning and logical deduction are essential components of class discussions and problem-solving activities, relying on the capacity to hold and manipulate multiple pieces of information simultaneously—a function closely tied to WM (Kyllonen & Christal, 1990). Research indicates that students with higher WMC often excel in tasks requiring the integration of prior knowledge with new information, facilitating more active engagement in complex cognitive activities (Alloway & Copello, 2013). In contrast, students with lower WMC may struggle to keep pace with fast-paced discussions, as they must retrieve and synthesize prior knowledge while maintaining attention to ongoing dialog (Alloway & Alloway, 2010). Structured discussion formats that reduce WM load, such as providing pre-discussion prompts or guided questioning, can enhance meaningful participation for all learners (Kyllonen & Christal, 1990).

#### 3.2.2. Note-Taking and Following Instructions

Note-taking is a cognitively demanding activity that relies heavily on WM. Effective note-taking requires students to process spoken information while summarizing key points in real time. Research indicates that as cognitive load increases, note-taking accuracy can decline, with students capturing fewer key details and struggling to organize their notes effectively. For instance, Peverly et al. (2013) found that handwriting speed, language comprehension, and WM were significantly related to the quality of notes taken during lectures. Similarly, following multi-step instructions requires learners to maintain the previous steps in WM while executing new ones, a process that individuals with WM challenges often find difficult. Strategies such as breaking tasks into simple steps and using clear, specific language can help support students with WM difficulties in following instructions.

#### 3.2.3. Classroom Management and Behavior Regulation

Self-regulation and classroom behavior are also likely influenced by WMC, as students with lower WMC tend to experience more difficulty sustaining attention, resisting distractions, and switching between tasks efficiently (Engle, 2018). These challenges are often linked to inattention and impulsivity, which can affect learners’ ability to manage classroom responsibilities and remain engaged with long-duration tasks. Classroom strategies that provide external cognitive supports, such as visual reminders, structured task transitions, and explicit sequencing of activities, can help mitigate these difficulties and enhance self-regulation in lower-WMC students.

Taken together, these findings underscore the fundamental role of WMC in both academic performance and classroom engagement. The evidence consistently highlights that students with higher WMC are better equipped to handle the cognitive demands of academic tasks and classroom activities, while those with lower WMC often face significant challenges. These findings suggest that instructional strategies aimed at reducing cognitive load and providing external support can help mitigate the difficulties faced by lower-WMC students, enhancing both their academic performance and classroom engagement. As research continues to explore the complex relationship between WMC and learning, it remains clear that fostering WMC through targeted interventions could have a profound impact on educational outcomes.

## 4. The ATI Framework in Educational Research

The previous sections established the role of WMC in academic performance. This section explores the ATI framework, a conceptual approach designed to examine how individual differences, particularly in cognitive aptitudes such as WMC, influence the effectiveness of instructional strategies. The ATI framework challenges the assumption that a single instructional strategy is universally effective. Instead, it emphasizes that the success of an instructional strategy depends on the learner’s cognitive characteristics, suggesting that tailoring instruction to individual aptitudes can optimize learning outcomes (Cronbach & Snow, 1977; Rohrer & Pashler, 2010).

### 4.1. The Evolution and Importance of the ATI Framework

Early ATI research focused primarily on broad intelligence measures, but recent work has shifted toward examining specific cognitive traits, such as WMC, executive function, and attentional control. These abilities provide a more precise explanation for why certain students benefit from particular instructional strategies while others struggle (Mayer, 2017). A growing body of research supports the role of WMC as a key aptitude in ATI studies. Unlike general intelligence, which encompasses multiple cognitive domains, WMC is a specific, measurable construct that directly influences how students encode, store, and retrieve information in real time. Empirical studies have confirmed that WMC moderates the effectiveness of instructional strategies such as multimedia learning (Sanchez & Wiley, 2009), retrieval practice (Tse & Pu, 2012), and spaced repetition (Bui et al., 2013). These studies demonstrate that instructional strategies optimized for lower-WMC learners, such as reducing extraneous cognitive load or providing structured guidance, do not necessarily hinder higher-WMC learners. This finding has important implications for designing equitable instructional strategies.

However, not all ATI effects are benign or additive. While many studies report ordinal interactions—in which an instructional strategy benefits one group more than another but does not harm either—a smaller but important set of studies demonstrates crossover interactions. In these cases, an instructional method that supports one group may actively impede learning in another. For instance, high levels of instructional guidance or scaffolding may benefit lower-WMC students but reduce performance among higher-WMC learners by limiting opportunities for productive struggle or deeper engagement (Kalyuga, 2007; Sweller, 2017). These crossover patterns are pedagogically significant, as they highlight situations where well-intentioned supports may unintentionally harm subsets of learners. Recognizing such effects is crucial for developing instructional approaches that are not only inclusive but also avoid inadvertently disadvantaging higher-capacity students.

As education continues to evolve toward technology-enhanced and adaptive learning environments, ATI is becoming increasingly relevant. Digital learning platforms now have the capacity to dynamically adjust instructional content based on students’ cognitive abilities, effectively implementing ATI principles in real-world settings (Schmidt-Weigand & Scheiter, 2011). By explicitly considering ATI effects, researchers and educators can develop more evidence-based, personalized learning strategies that accommodate a broad spectrum of cognitive strengths and weaknesses.

### 4.2. Methodological Approaches in ATI Research

To investigate how WMC interacts with instructional strategies, researchers use a variety of methodological approaches. These methods allow for the statistical detection of interactions between aptitude (WMC) and treatment (instructional intervention), helping to determine whether certain instructional strategies benefit some learners more than others.

One of the most widely used techniques in ATI research is moderation analysis, which tests whether the relationship between an instructional strategy and learning outcome changes depending on WMC. For example, Sanchez and Wiley (2006) examined how extraneous visual information in multimedia learning affected high- and low-WMC learners. Using moderation analysis, they found that lower-WMC learners were significantly more distracted by irrelevant images, whereas higher-WMC learners were unaffected. This suggests that removing unnecessary visuals in educational materials benefits lower-WMC learners without harming others, a clear demonstration of an ATI effect. Similarly, Agarwal et al. (2017) applied moderation analyses to study retrieval practice, finding that lower-WMC learners benefited disproportionately from testing when feedback was provided, whereas higher-WMC learners showed more stable performance across conditions. These findings highlight the importance of considering WMC when designing instructional strategies that rely on retrieval practice.

While moderation analyses are effective for detecting interaction effects, they often require researchers to dichotomize continuous traits like WMC into “high” or “low” categories. This not only reduces statistical power but also oversimplifies the nature of individual differences. A more advanced approach involves structural equation modeling (SEM) and latent variable interaction models, which preserve the continuous nature of cognitive traits and account for measurement error in both aptitude and outcome variables. By modeling WMC as a latent construct based on multiple observed indicators, SEM provides more precise estimates of aptitude–treatment interactions than traditional moderation techniques.

Latent variable interaction models extend this precision further by capturing the subtle ways in which individual differences in WMC influence responsiveness to instruction. These models allow researchers to test whether the effect of an instructional strategy varies across the full range of WMC, rather than relying on arbitrary groupings. For instance, recent work has shown that learners with high attentional control—a construct often correlated with WMC—may not benefit from the same supports (e.g., scaffolding or retrieval cues) that assist learners with lower capacity (Unsworth et al., 2021). Although statistically complex, SEM and latent variable approaches offer richer, more accurate representations of cognitive aptitude, making them essential tools for developing personalized, empirically grounded instructional strategies.

### 4.3. Practical Considerations in ATI Research

While ATI research provides valuable insights into cognitive learning processes, several methodological and practical considerations must be addressed. One key challenge is ensuring the accurate measurement of WMC, as not all WM tasks capture the same cognitive processes. Many ATI studies rely on complex span tasks, such as operation span, reading span, and symmetry span, to assess WMC. While these tasks are reliable measures of WMC, some, like operation span, are more domain-general and may not be as effective at predicting specific academic outcomes, such as reading comprehension. For example, the reading span task, which involves holding and integrating verbal information, is better suited for predicting reading comprehension performance. Furthermore, some simple span tasks, like digit span, do not measure attentional control, a crucial component of WMC (Redick et al., 2016). Researchers must carefully select validated WM assessments that align with the specific cognitive processes relevant to their study in order to ensure the precise measurement of aptitude differences.

Another challenge in ATI research is accounting for additional factors that influence instructional effectiveness, such as prior knowledge, motivation, and self-regulation skills. Failing to control for these variables can lead to spurious ATI effects. For example, a student with low WMC but high motivation may compensate for WM deficits by employing greater effort and engagement, potentially masking the true interactions between WMC and instructional strategies (Sweller, 2017). Similarly, a student with low WMC but strong self-regulation skills might manage cognitive demands more effectively than a student with similarly low WMC but poor self-regulation, thus obscuring the real relationship between WMC and instructional outcomes (Duckworth & Seligman, 2005). To provide a clearer understanding of how WMC interacts with instructional strategies, future research should carefully control for these confounding variables to more accurately assess their impact on learning.

Many ATI studies are conducted in controlled laboratory environments rather than authentic educational settings. While these experiments provide valuable theoretical insights, their real-world applicability remains limited. Future research should investigate how ATI effects translate to classroom settings, online learning environments, and collaborative learning scenarios to enhance practical relevance. Additionally, most ATI studies treat WMC as a stable, trait-level characteristic, but emerging research suggests that WMC can fluctuate based on factors like fatigue, stress, and cognitive load (Ilkowska & Engle, 2010). Exploring whether temporary changes in WMC influence ATI effects would offer a more dynamic understanding of how instructional treatments interact with cognitive capacity.

In summary, the ATI framework offers a compelling model for understanding how instructional strategies interact with individual differences in WMC. By employing rigorous methodological techniques, researchers can gain deeper insights into how ATI effects shape learning outcomes. However, to maximize the real-world applicability of these findings, future studies should consider state-dependent changes in WMC, explore instructional interactions in authentic educational contexts, and refine methods for adaptive instructional design. Understanding how WMC moderates instructional effectiveness is crucial for developing more inclusive, evidence-based educational practices that accommodate diverse learners.

## 5. WM and Instructional Strategies: An ATI Perspective

Understanding how instructional strategies interact with WMC is central to optimizing learning outcomes for diverse student populations. While effective instructional strategies have been identified through cognitive and educational research, their impact often depends on the learner’s cognitive abilities. The ATI framework provides a lens through which to examine how different instructional strategies benefit students based on their WMC. This section discusses key instructional approaches, reviewing how they interact with WMC and providing evidence-based recommendations for educational practice.

### 5.1. Multimedia Learning

Multimedia instruction—integrating verbal, visual, and auditory materials—places considerable demands on WM, as learners must coordinate multiple streams of information. According to the cognitive load theory (CLT; Sweller, 1988), multimedia materials can either facilitate or hinder learning, depending on how they interact with WMC. Studies indicate that lower-WMC learners are particularly vulnerable to extraneous cognitive load in multimedia learning environments. Sanchez and Wiley (2006) found that unnecessary visuals impaired comprehension for lower-WMC learners but had minimal effect on higher-WMC learners. Similarly, Lusk et al. (2009) demonstrated that segmenting multimedia presentations benefited lower-WMC individuals by reducing information overload. To optimize multimedia learning, instructional designers should limit extraneous elements, such as decorative images or animations, and provide segmentation, allowing students to control the pace of content delivery. Additionally, dual-channel processing—where spoken words are paired with relevant visuals instead of redundant on-screen text—has been shown to reduce cognitive demands for lower-WMC learners while maintaining benefits for higher-WMC learners (Mayer & Moreno, 2003).

While this review centers on ATIs, many of the patterns described here are consistent with CLT. For example, interventions that benefit lower-WMC learners—such as reducing visual clutter or enabling self-paced navigation—function by reducing extraneous cognitive load. This alignment suggests that CLT offers a complementary perspective on why certain instructional strategies interact with learner aptitudes, particularly in contexts where managing multiple information sources taxes limited cognitive resources.

### 5.2. Test-Enhanced Learning (Retrieval Practice)

The retrieval practice effect—the process of recalling learned information to strengthen memory—has been widely recognized as one of the most effective learning strategies (Roediger & Butler, 2011). However, research suggests that WMC moderates the benefits of retrieval practice, particularly in high-stakes or anxiety-inducing learning contexts. Tse and Pu (2012) found that while retrieval practice benefited all learners, the effectiveness of repeated testing was less pronounced for individuals with lower WMC and higher test anxiety. Specifically, lower-WMC learners with high test anxiety showed more errors and less benefit from testing compared to higher-WMC learners. Brewer and Unsworth (2012) found that retrieval practice benefits learners regardless of WMC, but that lower-ability learners may experience greater overall gains due to their poorer baseline memory performance. Agarwal et al. (2017) further demonstrated that retrieval practice is especially beneficial for lower-WMC learners when cognitive load is minimized. These findings suggest that retrieval practice should be structured to reduce cognitive demands for lower-WMC learners, particularly in contexts that may induce test anxiety, by incorporating low-stakes quizzes, cumulative assessments, and immediate feedback.

### 5.3. Spaced Learning (Distributed Practice)

The spacing effect—the phenomenon where information retention improves when learning is distributed over time rather than massed into a single session—has been extensively studied in cognitive psychology (Cepeda et al., 2006). Research suggests that the optimal spacing interval may depend on individual differences such as working memory capacity (WMC). For instance, Bui et al. (2013) found that individuals with higher WMC benefited more from difficult intervening tasks between repetitions, while those with lower WMC showed better retention with easier intervening tasks. This indicates that lower-WMC learners may struggle with complex tasks during longer intervals due to difficulties in managing cognitive load. Similarly, Delaney et al. (2017) examined whether WMC influences the spacing effect in cued recall and found that while both WMC and spacing independently improved memory performance, they did not significantly interact—suggesting that spacing benefits all learners regardless of WMC. To optimize spaced learning, educators should consider adjusting the difficulty of intervening tasks based on individual WMC levels. Adaptive learning platforms can incorporate dynamic scheduling that tailors task complexity and spacing intervals to match learners’ cognitive capacities, thereby enhancing learning outcomes.

### 5.4. Interleaved Practice

The interleaving effect—alternating between different types of problems or concepts—has been shown to enhance long-term retention and problem-solving skills compared to blocked practice (Rohrer & Taylor, 2007). However, its effectiveness varies based on WMC. Sana et al. (2018) directly investigated whether WMC moderates the interleaving effect and found that interleaved practice benefits learners regardless of their WMC, suggesting that its advantages are broadly applicable. Similarly, Guzman-Munoz (2016) examined how WMC influences the effectiveness of interleaved inductive learning and found that while interleaving benefits all learners, those with higher WMC may find it easier to process the mixed examples. Suzuki et al. (2020) also explored WMC’s role in proceduralizing L2 syntax through interleaved grammar practice, suggesting that cognitive resources influence the effectiveness of interleaving in certain contexts. Additionally, Sana et al. (2017) demonstrated that study sequence impacts inductive learning of cognitive concepts, further supporting the instructional value of interleaved practice. Given these findings, educators should integrate interleaved practice into instructional design, particularly in ways that promote conceptual differentiation and retrieval-based learning. Structured worksheets, mixed-problem assignments, and varied practice sessions can help ensure that all students benefit from this evidence-based strategy.

### 5.5. Worked Examples and Explicit Instruction

Worked examples—step-by-step demonstrations of problem-solving procedures—are particularly beneficial for learners with lower working memory capacity (WMC), as they reduce cognitive load by minimizing the need for simultaneous problem-solving and information retention (Sweller et al., 2019). According to CLT, worked examples effectively manage cognitive demands, especially for novice learners. Research indicates that lower-WMC learners benefit more from worked examples compared to unstructured problem-solving approaches, as worked examples help scaffold complex information and prevent cognitive overload (Sweller, 2017; Paas & van Merriënboer, 1994). The expertise reversal effect further suggests that as learners develop proficiency, the effectiveness of worked examples diminishes, allowing higher-WMC learners to transition to independent problem-solving earlier without negative effects (Kalyuga, 2007). Similarly, Renkl (2014a, 2014b) highlights that gradually fading worked examples—transitioning from fully guided solutions to problem-solving tasks—is an effective instructional strategy that accommodates learners as they develop expertise. For instructional application, educators should consider this gradual fading process to ensure that lower-WMC learners are not overwhelmed while still promoting active engagement.

### 5.6. Instructional Guidance: Inquiry vs. Direct Instruction

The debate between inquiry-based learning and direct instruction is particularly relevant in the context of working memory (WM). While higher-WMC learners can often manage the open-ended nature of inquiry-based learning, lower-WMC learners may struggle with the cognitive demands of self-directed discovery. Kirschner et al. (2006) found that lower-WMC students benefited significantly more from explicit instruction compared to inquiry-based approaches, as direct guidance reduces extraneous cognitive load. Similarly, Ginns (2005) highlighted that structured guidance, such as worked examples, helps learners better retain information by managing cognitive load effectively. However, well-scaffolded inquiry learning, where guidance is gradually removed as learners develop expertise, can mitigate WM-related challenges. To optimize learning, educators should provide structured guidance early on and then gradually transition to inquiry-based methods as students build expertise.

### 5.7. Flipped Classrooms

Flipped classrooms—where students engage with instructional materials before class and apply concepts during in-class activities—can influence cognitive load and accommodate different WMC levels. Diningrat et al. (2023) investigated the effect of an extended flipped classroom model on students’ reading comprehension and found that while flipped learning was beneficial, students with high WMC outperformed those with lower WMC. This suggests that flipped classrooms can be effective but must be carefully structured to support lower-WMC learners. Bergmann and Sams (2012) emphasized that flipped classrooms reduce cognitive load by allowing students to learn at their own pace, which may be particularly helpful for learners who struggle with memory retention. However, to maximize effectiveness, flipped learning must ensure that students complete pre-class work and receive structured guidance during in-class activities.

### 5.8. Feedback (Types and Delivery)

Feedback plays a crucial role in learning, but its effectiveness depends on WMC. Li (2013) investigated the interactions between implicit and explicit feedback and individual differences in language analytic ability and WM in second language learning. The study found that learners with higher WMC benefited more from explicit feedback, while those with lower WMC showed comparable gains from both implicit and explicit feedback. Similarly, Fyfe et al. (2015) examined the role of WMC in the effectiveness of different feedback types in mathematical problem-solving. Their research indicated that lower-WMC learners benefited more from outcome feedback, which provides information on the correctness of an answer, whereas higher-WMC learners benefited similarly from both outcome and strategy feedback, the latter offering guidance on problem-solving strategies. These findings are consistent with the broader feedback literature, which suggests that different types of feedback can optimize learning based on cognitive capacity (Hattie & Timperley, 2007). To optimize learning, feedback should be tailored based on cognitive load considerations, providing structured hints and scaffolding for lower-WMC learners while allowing more open-ended correction for higher-WMC students.

In summary, this section has highlighted how WMC may influence the effectiveness of different instructional strategies. While direct empirical studies on WMC–instructional strategy interactions remain limited, findings from CLT and related research suggest that lower-WMC learners often benefit from structured, scaffolded, and guided approaches that help manage cognitive demands. In contrast, higher-WMC learners may be better equipped to engage with more autonomous and generative learning environments that require greater self-regulation and cognitive flexibility. However, given the limited number of studies directly examining these interactions, further research is needed to refine our understanding of how WMC shapes learning outcomes across different instructional contexts. The following section will explore the interplay between metacognition, retrieval cues, and WMC, considering how learning strategies can be adapted to accommodate individual cognitive differences and optimize retention and transfer.

## 6. WM, Metacognition, and Retrieval Cues

While instructional treatments can be designed to accommodate differences in WMC, another critical factor influencing learning is metacognition—the ability to monitor and regulate one’s own cognitive processes. Metacognitive skills enable learners to assess their understanding, adjust their study strategies, and retrieve relevant information efficiently. However, the effectiveness of metacognitive strategies is closely tied to WMC, as individuals with lower WMC often struggle with self-monitoring and controlled retrieval processes. This section explores the relationship between WMC, metacognition, and retrieval cues, offering insights into how instructional design can better support learners with varying WMC.

### 6.1. Metacognitive Monitoring and Strategies

Metacognitive monitoring—the ability to evaluate one’s level of understanding and adjust learning strategies accordingly—is essential for academic success. However, research suggests that lower-WMC learners tend to be less accurate in monitoring their comprehension and predicting their performance compared to higher-WMC learners (Griffin et al., 2008). This discrepancy arises because effective metacognitive monitoring requires holding multiple pieces of information in WM while simultaneously evaluating their coherence and accuracy, a process that is particularly demanding for those with limited WM resources. Griffin et al. (2008) found that lower-WMC learners often overestimate their comprehension and fail to recognize gaps in their understanding. However, this monitoring deficit can be mitigated through structured metacognitive prompts. When participants were prompted to self-explain key concepts after reading, their comprehension monitoring accuracy improved, effectively reducing WMC-related performance gaps. Similarly, rereading the text before making comprehension judgments helped lower-WMC learners improve their monitoring accuracy, suggesting that reducing concurrent WM demands allows for more accurate self-assessment. These findings are consistent with the broader metacognitive literature, which suggests that students with higher WMC tend to have better metacognitive awareness and strategy adjustment abilities (Koriat, 2007; Veenman et al., 2006).

From an instructional standpoint, embedding metacognitive scaffolds can enhance self-monitoring skills for lower-WMC learners. Strategies such as prompting learners to generate explanations, summarize key points, or predict test performance can help them regulate their learning more effectively. Educators can also train students in metacognitive strategies explicitly, teaching them to pause periodically, assess their understanding, and adjust their study methods accordingly. It is possible that self-regulated learning (SRL) training disproportionately benefits lower-WMC learners. By incorporating goal-setting, self-questioning, and monitoring strategies, lower-WMC individuals can develop compensatory mechanisms that allow them to allocate cognitive resources more effectively. This suggests that explicit instruction in metacognitive regulation should be integrated into curricula, particularly for students who struggle with attentional control and self-monitoring. These findings are echoed by studies on cognitive load and self-regulation, suggesting that explicitly teaching metacognitive strategies can mitigate the cognitive challenges faced by lower-WMC learners (e.g., Dunlosky & Metcalfe, 2009; Schraw, 2006).

### 6.2. Retrieval Cues and Search Processes in WM

Retrieval cue selection is a critical mechanism that influences learning efficiency. WM plays a pivotal role in guiding controlled searches of Long-Term Memory (LTM), but lower-WMC learners often struggle with selecting appropriate retrieval cues, leading to increased interference from irrelevant information (Unsworth & Engle, 2007). Unsworth et al. (2012a) found that lower-WMC individuals were more likely to experience retrieval failure due to ineffective cue selection. However, providing structured retrieval cues, such as category labels, contextual hints, or partial word cues, significantly improved recall performance for these learners. This suggests that educators should incorporate retrieval scaffolding into learning materials to help lower-WMC students retrieve information more efficiently.

Classroom assessments can be designed to support retrieval for lower-WMC learners by incorporating scaffolded recall questions with keywords or conceptual categories to reduce retrieval failures. Concept maps and structured outlines can also serve as external retrieval cues, helping students organize and recall information. Additionally, cumulative assessments that revisit prior concepts can reinforce memory recall pathways and make retrieval a habitual process. In fact, lower-WMC learners seem to benefit from frequent low-stakes quizzes with guided retrieval cues, which strengthen recall pathways and reduce reliance on inefficient search strategies (Agarwal et al., 2017; Unsworth & Engle, 2007). This aligns with research on spaced retrieval practice, suggesting that gradually introduced retrieval cues can help build strong memory networks for lower-WMC learners.

Tailoring instructional strategies to support metacognitive and retrieval processes is crucial for optimizing learning outcomes. By incorporating techniques like metacognitive training, structured retrieval cues, and spaced retrieval practice, educators can better support lower-WMC learners and bridge performance gaps. Continued research into adaptive strategies will further enhance personalized learning, helping students of all cognitive abilities reach their full potential.

## 7. Integrating ATI Research into Educational Practice

The ATI framework provides valuable insights into how instructional strategies can be tailored to accommodate individual differences in WMC. While ATI research has traditionally been conducted in controlled laboratory settings, the challenge now lies in translating these findings into real-world educational applications. This section explores practical approaches for implementing ATI-based strategies in classroom instruction, assessment, and adaptive learning environments while addressing key challenges and research considerations for future investigations.

### 7.1. Guidelines for Educators

One of the most effective ways to integrate ATI principles into education is through adaptive instruction, in which instructional content dynamically adjusts based on learners’ cognitive characteristics. Advances in digital learning technologies and intelligent tutoring systems allow for the real-time assessment of individual cognitive abilities, enabling instructional materials to be personalized accordingly. For example, adaptive learning platforms can measure students’ WMC using embedded cognitive tasks and then modify instructional complexity based on their processing capacity. Research suggests that such adaptive learning environments improve engagement and retention by ensuring that learners are neither overwhelmed nor under-stimulated (Kalyuga & Plass, 2017; Paas & van Merriënboer, 2020). Higher-WMC learners can be presented with more complex, self-guided challenges, while lower-WMC learners receive structured, scaffolded instruction to manage cognitive load effectively (Kalyuga, 2009).

Differentiated instruction provides another avenue for applying ATI principles in classrooms. Unlike adaptive learning, which relies on automated adjustments, differentiated instruction requires teachers to proactively tailor lesson plans, assessments, and instructional strategies to meet diverse cognitive needs. Educators can use informal assessments of WMC—such as students’ ability to follow multi-step instructions, maintain attention, and recall previously learned material—to determine which students require additional cognitive support. For lower-WMC learners, instructional strategies should emphasize breaking down complex tasks into smaller, more manageable steps, incorporating guided practice with worked examples, and providing external memory aids (Swanson et al., 2013). In contrast, higher-WMC learners tend to benefit from instructional techniques that encourage independent problem-solving, such as inquiry-based learning, interleaved practice, and self-explanation (Van Merriënboer & Sweller, 2010).

The ATI framework also aligns with universal design for learning (UDL), an educational approach that promotes accessibility for all students by offering multiple means of engagement, representation, and expression. By integrating principles of the cognitive load theory, UDL allows students with varying WMC levels to learn through different modalities without being segregated into separate instructional tracks (Meyer et al., 2014). For example, multimedia instruction can incorporate both text-based and auditory explanations, enabling students to select the modality that best suits their cognitive processing capacity (Mayer, 2017). Similarly, multiple formats for demonstrating knowledge—such as written responses, verbal explanations, or concept mapping—can help lower-WMC learners express their understanding more effectively.

A crucial pedagogical strategy within ATI-informed instruction is scaffolding, which provides structured support at the beginning of the learning process and gradually reduces assistance as students gain proficiency. Scaffolding techniques, including guided questioning, explicit modeling, and structured problem breakdowns, help lower-WMC learners manage cognitive demands more effectively (Van de Pol et al., 2010). Research suggests that lower-WMC learners struggle with open-ended problem-solving tasks due to increased cognitive load, whereas structured guidance helps them acquire and apply new information more efficiently (Kirschner et al., 2006). In contrast, higher-WMC learners often benefit from reduced instructional guidance, as they can engage in deeper cognitive processing and self-directed learning (Clark et al., 2012). Therefore, educators should gradually transition students from explicit instruction to independent problem-solving, ensuring that instructional demands align with individual cognitive capacities.

#### 7.1.1. Assessment and Classroom Collaboration in ATI-Based Instruction

Assessment design plays a critical role in ensuring that ATI principles are applied effectively. Traditional assessments often assume that all students process and retrieve information similarly, yet research suggests that WMC influences test performance independently of actual content knowledge (Unsworth & Robison, 2020). To mitigate this, assessments should be structured to minimize unnecessary WM demands for lower-WMC learners. For example, cued recall questions, which provide prompts or structured retrieval cues, can help students access relevant information without overwhelming their cognitive resources (Unsworth et al., 2012a). Similarly, extended testing time or structured cognitive breaks can enhance performance by reducing stress-induced WM depletion (Alloway & Alloway, 2010). In contrast, higher-WMC learners can be challenged with complex, integrative assessments that require synthesizing information across multiple domains (Tarchi et al., 2024).

Classroom collaboration and peer-assisted learning also offer opportunities for ATI-informed instruction. Research has shown that structured peer collaboration benefits lower-WMC learners by distributing cognitive demands among multiple individuals, effectively reducing the cognitive load on any single learner (Kirschner et al., 2018). However, collaborative learning environments must be carefully structured to ensure that lower-WMC learners actively contribute rather than passively relying on higher-WMC peers. Techniques such as reciprocal teaching, where students take turns explaining concepts, and jigsaw learning, where each student is responsible for mastering a different subtopic before teaching it to peers, can promote engagement while balancing cognitive demands (Palincsar & Brown, 1984; Rosenshine & Meister, 1994).

#### 7.1.2. Adapting Group Instruction Without Stigmatization

While adaptive and differentiated instruction are well suited to individualized digital learning environments, many educational settings rely on group-based instruction. Applying ATI principles in a classroom context without stigmatizing students with lower WMC requires a multi-faceted approach. One effective method is to incorporate universal design for learning (UDL), which provides multiple means of engagement, representation, and expression. For instance, a lesson can simultaneously include verbal explanations, visual diagrams, and interactive activities, allowing students to select the modality that best aligns with their cognitive processing capacity. Such an approach ensures that learners benefit from differentiated supports without being visibly labeled.

In addition to UDL, flexible grouping strategies can help mitigate stigmatization. Rather than categorizing students by ability, educators can adopt dynamic grouping, where students are periodically reorganized based on task requirements or instructional goals. This approach promotes collaborative learning without permanently isolating lower-WMC learners. For example, in problem-solving tasks, groups can be intentionally composed of diverse learners, enabling peer support without overtly highlighting individual differences.

Layered scaffolding offers another practical solution. Rather than explicitly categorizing students, educators can make optional supports universally available, such as guided practice, visual cues, or step-by-step instructions. Lower-WMC learners can choose to access these supports without being singled out, while higher-WMC learners can engage in independent problem-solving. This self-selected support model allows all students to engage at an appropriate cognitive level without stigma.

Finally, instructor sensitivity and professional development are critical. Educators should be trained to recognize diverse cognitive needs without relying on explicit labels or making assumptions. By adopting a strength-based perspective—emphasizing that some learners benefit from structured practice while others thrive with exploratory tasks—teachers can maintain an inclusive, respectful classroom culture. Collectively, these strategies ensure that ATI principles can be effectively implemented in group instruction without risking negative social perceptions, allowing all learners to benefit from cognitively aligned support.

### 7.2. Guidelines for Researchers

While Section 7.1 focused on how educators can apply ATI principles in instructional settings, this section offers complementary guidance for researchers. We highlight methodological and design considerations for studying aptitude–treatment interactions and suggest empirical strategies to improve the rigor and ecological validity of future research.

From a research perspective, the ATI framework presents an opportunity to refine methodologies for examining how cognitive factors influence learning. However, many existing studies lack the statistical rigor required to fully capture these interactions. Moderation analyses remain the most commonly used method for testing ATI effects, allowing researchers to assess how WMC influences the effectiveness of various instructional strategies. Structural equation modeling (SEM) and latent variable interaction models offer additional statistical tools that can provide a more precise understanding of how cognitive traits moderate learning outcomes across different instructional conditions.

Practical considerations must also be taken into account when measuring WMC in ATI research. Traditional WM tasks, such as complex span tasks, remain the gold standard for assessing WMC, but researchers must ensure that these measures are both reliable and ecologically valid in instructional contexts. Many studies rely on laboratory-based assessments, but these tasks may not fully capture how WMC operates in real-world educational settings (Holmes & Gathercole, 2014). Additionally, small sample sizes can obscure ATI effects, as these interactions are often subtle. To enhance statistical power, studies should be designed with sufficient sample sizes and robust analytic techniques to detect meaningful interactions.

Moreover, longitudinal research is needed to determine the sustained impact of ATI-based instructional approaches. Most ATI studies examine short-term learning gains, but tracking students’ cognitive performance over time would provide more robust evidence of how WMC interacts with instructional treatments across different stages of learning. Longitudinal designs would also allow researchers to investigate whether repeated exposure to ATI-aligned instruction can help lower-WMC learners develop compensatory strategies, potentially reducing performance gaps over time.

A key challenge in ATI research is ensuring ecological validity, as many studies are conducted in controlled laboratory settings rather than real-world educational environments. Future research should prioritize classroom-based interventions, examining how WMC interacts with instructional strategies in authentic learning environments. Additionally, expanding ATI research beyond traditional academic domains—such as mathematics and reading comprehension—to emerging instructional paradigms like flipped classrooms, virtual learning environments, and AI-driven tutoring systems will provide a more comprehensive understanding of how ATI principles apply in diverse educational settings.

Another major limitation of current ATI research is the lack of direct cognitive assessments in classroom settings. While standardized WM tasks such as complex span tasks are used in research, they are not commonly integrated into everyday educational practice. Instead, educators must rely on indirect indicators of WMC, such as attention regulation, task persistence, and instructional responsiveness (Gathercole & Alloway, 2008). Developing efficient, classroom-friendly cognitive assessment tools would allow for more precise tailoring of instructional strategies based on individual cognitive capacities.

The ATI framework offers a powerful model for designing instructional strategies that align with students’ cognitive abilities, particularly WMC. By incorporating adaptive learning, differentiated instruction, scaffolding, and structured assessment design, educators can create more equitable and effective learning environments. While challenges remain in scaling ATI-based instruction, advances in educational technology and adaptive learning models provide promising pathways for widespread implementation. Future research should continue refining statistical methodologies, expanding ATI investigations across different instructional contexts, and exploring interventions aimed at enhancing WMC. As the field progresses, bridging the gap between cognitive science and educational practice will be essential in ensuring that ATI-based strategies translate into meaningful improvements in student learning outcomes.

## 8. Future Directions for ATI and WM in Education

In addition to methodological recommendations, there are several conceptual and applied frontiers that merit further investigation. This final section outlines unresolved questions and proposes future research directions spanning instructional strategy design, cognitive states, learner diversity, and emerging educational technologies.

Despite significant progress in understanding how WMC interacts with instructional strategies, many questions remain unresolved. The ATI framework has provided valuable insights, but further research is needed to refine our understanding of how cognitive traits shape learning outcomes. This section highlights key areas for future investigation, including under-explored instructional strategies, contextual factors influencing WMC, and the implications of ATI research for special populations.

One of the most pressing gaps in ATI research is the limited investigation of analogy learning and its interaction with WMC. Analogies are powerful instructional tools that facilitate knowledge transfer by mapping familiar concepts onto new material, aiding conceptual understanding in subjects like science, mathematics, and second language acquisition (Richland et al., 2007). However, it remains unclear whether the cognitive demands of analogy learning differentially impact students based on their WMC. On one hand, analogies may reduce cognitive load for lower-WMC learners by providing structured knowledge frameworks, but on the other hand, constructing and applying analogies requires abstract reasoning and relational integration, which may disproportionately burden learners with lower WMC (Richland & Burchinal, 2013). Future research should explore how different types of analogies—simple, complex, self-generated, or instructor-provided—affect learners across the WMC spectrum.

Similarly, spaced learning interventions require further refinement within an ATI framework. While spacing has been consistently shown to enhance retention (Cepeda et al., 2006; Roediger & Butler, 2011), the optimal spacing interval for learners with varying WMC remains an open question. Some evidence suggests that higher-WMC learners benefit from longer intervals between study sessions due to their superior ability to retrieve and integrate previously learned information, whereas lower-WMC learners may require shorter, more frequent exposures to prevent information loss (Bui et al., 2013). However, current research is largely correlational, and few studies have directly manipulated spacing conditions based on individual WMC. Future research should systematically examine whether adjusting spacing intervals based on WMC leads to more effective retention and knowledge transfer.

Another promising but controversial area of research is WM training and its potential impact on learning outcomes. While early studies suggested that WM training could lead to broad cognitive improvements (Jaeggi et al., 2008), more recent meta-analyses indicate that such gains are typically limited to the trained task, with minimal transfer to academic performance or real-world problem-solving (Melby-Lervåg et al., 2016). However, it remains unclear whether specific populations—such as students with WM deficits or learning disabilities—might derive greater benefits from targeted training interventions. Future research should explore whether combining WM training with ATI-informed instructional techniques could produce more meaningful improvements in learning outcomes, particularly for at-risk student populations.

Beyond instructional techniques, contextual and situational factors play a crucial role in moderating WMC’s influence on learning. While WMC is generally considered a stable trait, evidence suggests it can fluctuate based on stress, fatigue, anxiety, and environmental distractions (Ilkowska & Engle, 2010). This raises important questions about whether ATI-based instructional strategies should be adapted based on learners’ transient cognitive states. For example, learners with high WMC may perform well under optimal conditions but experience cognitive overload when faced with high-stakes testing or information-dense lectures (Vogel & Schwabe, 2016). Conversely, lower-WMC learners may benefit from interventions that minimize situational stressors, such as structured retrieval practice or externally provided organizational cues (Putnam et al., 2016). More research is needed to determine how state-dependent variations in WMC interact with instructional treatments and how educators can accommodate these fluctuations.

Developmental factors must also be considered. WMC changes significantly over the lifespan, with younger learners typically showing lower and more variable capacity. These developmental differences suggest that ATI effects may be particularly pronounced in childhood, and instructional strategies may need to be adapted accordingly. For example, younger learners may benefit from more sustained scaffolding, whereas older learners may benefit from the gradual removal of supports.

Another particularly important but underdeveloped area of ATI research concerns special populations, including students with learning disabilities, ADHD, and neurological disorders. Many ATI studies focus on general populations, yet learners with specific cognitive impairments may exhibit fundamentally different interactions with instructional strategies (Gathercole & Alloway, 2008). For example, students with ADHD often exhibit reduced WMC and impaired attentional control, which may alter the effectiveness of interventions designed to support lower-WMC learners (Martinussen & Major, 2011). Similarly, students with dyslexia may struggle with instructional methods that rely heavily on phonological WM, whereas students with autism spectrum disorder (ASD) may show differential responses to structured versus open-ended instructional designs (Pellicano, 2012). Tailoring ATI-based strategies to meet the needs of diverse learners is a critical next step for research in this area.

Another important area for future research is the role of motivation and self-regulated learning strategies within ATI-based instruction. Students with lower WMC often face additional challenges related to task persistence, effort allocation, and self-monitoring, which can influence how well they respond to instructional strategies designed to accommodate cognitive load differences (Dunlosky & Thiede, 2013). Metacognitive training—including techniques such as self-explanation, reflection prompts, and guided goal-setting—may serve as a buffer against cognitive overload by helping lower-WMC learners develop compensatory strategies (Bjork et al., 2013). Future studies should investigate how motivational factors interact with cognitive aptitudes in shaping learning outcomes, particularly in self-paced learning environments where students must regulate their own study habits.

Future research should also consider the role of technology and adaptive learning environments in delivering ATI-informed instruction. With the rise in artificial intelligence-driven educational platforms, there is an opportunity to develop adaptive systems that dynamically adjust instructional complexity based on real-time assessments of learners’ cognitive capacity (Koedinger et al., 2012). Such systems could personalize learning experiences by modulating cognitive load, providing targeted scaffolding, or recommending optimal retrieval practice intervals (Kalyuga, 2007). However, rigorous empirical validation is necessary to determine the effectiveness of these approaches across different learner populations.

## 9. Conclusions

This paper has explored the critical role of WMC in shaping how learners respond to instructional treatments, emphasizing the importance of integrating the ATI framework into educational research and practice. Decades of research in cognitive psychology and education have established that individual differences in WMC significantly influence learning outcomes across a wide range of academic domains, including reading comprehension, mathematics, second language acquisition, and science reasoning (Sweller, 2017; Unsworth et al., 2021). These findings underscore the necessity of moving beyond one-size-fits-all instructional approaches and instead tailoring teaching methods to align with learners’ cognitive abilities.

Beyond its implications for educators, this research also highlights important considerations for researchers designing future ATI studies. Future research should focus on how ATI principles can be leveraged in modern, technology-enhanced learning environments, including digital classrooms, virtual reality-based training, and AI-powered adaptive tutoring systems. Investigating how ATI principles can be incorporated into emerging technologies will be crucial for advancing the practical applicability of this research.

Ultimately, this paper underscores the need for continued research and the practical application of ATI principles in education. By systematically considering how WMC influences learning, both researchers and educators can contribute to a more nuanced and effective understanding of how instructional strategies can be tailored to meet the needs of all learners. The goal is not simply to identify which methods work best but to understand for whom and under what conditions they work best, ensuring that education becomes a truly personalized and impactful experience for every student.

## Data Availability

No new data were collected.

## Abbreviations

The following abbreviations are used in this manuscript:

ATI	aptitude–treatment interaction
WM	working memory
WMC	working memory capacity
